# Urothelial carcinoma of the upper urinary tract diagnosed via *FGFR3* mutation detection in urine: a case report

**DOI:** 10.1186/1471-2490-12-20

**Published:** 2012-08-08

**Authors:** Daniel M Silverberg

**Affiliations:** 1 5018 Medical Center Circle, Suite 240, Allentown, PA 18106, USA

**Keywords:** Cancer, Ureter, Renal pelvis, *FGFR3*, PCR, Kidney, Bladder, Urothelial carcinoma, Diagnosis, CertNDx

## Abstract

**Background:**

Upper urinary tract cancer is typically diagnosed with urine cytology and imaging techniques. These assays can be limited by sensitivity, specificity, or technical issues making some diagnoses difficult.

**Case presentation:**

A 73-year old man presented to the clinic with a right renal pelvis filling defect that was detected by a CT-scan performed for unrelated reasons. Urine cytology was negative. Cystoscopy, retrograde pyelogram, and partial ureteroscopy were unable to visualize the lesion resulting in an indeterminate diagnosis. A subsequent CT scan confirmed the renal lesion which appeared to have become larger and was consistent with urothelial carcinoma. A urine based genetic assay was used to test for the presence of urothelial carcinoma. This assay evaluates the presence of mutations in fibroblast growth factor receptor 3 (*FGFR3*). Mutations in *FGFR3* are known to be associated with urothelial carcinoma and have a positive predictive value of 95% when detected in patients with no history of TCC. A mutation in exon 10 (Y375C) of *FGFR3* was identified. Nephroureterectomy was performed and the subsequent pathology confirmed urothelial carcinoma. In addition, PCR analysis on isolated tumor tissue indicated the tumor carried the same *FGFR3* mutation as that of the DNA isolated from urine, consistent with the tumor being the origin of the mutant DNA.

**Conclusion:**

This study indicates that the *FGFR3* urine assay, which was originally developed to monitor bladder cancer, is also a useful tool for diagnosing upper urinary tract cancer in a real-life setting.

## Background

Urothelial carcinoma is the fourth most common cancer and can be located in the lower or upper urinary tract. Upper urinary tract carcinomas, which are nearly all urothelial carcinomas, are uncommon and account for only 5–10% of all urinary carcinomas [1,2].

Diagnosis of upper urinary tract cancer has traditionally depended on urine cytology and imaging techniques including CT urography, retrograde pyelography and direct pyeloscopy [2,3]. These assays are all useful but individually can be limited by sensitivity, specificity, or technical issues. In fact, the American Urologic Association and European Association of Urology recommend the use of multiple approaches for diagnosing possible upper urinary tract malignancies [2,4].

Activating mutations in the fibroblast growth factor receptor 3 (*FGFR3*) occur in >50% of low-grade and low-stage bladder tumors (about 64–85% of pTa tumors carry *FGFR3* mutations) [5-9]. Eight common *FGFR3* mutations in 3 exons (exons 7, 10, and 15) are associated with >90% of all known mutant *FGFR3* positive bladder cancers. Several urine-based genetic assays have been developed to detect *FGFR3* mutations in patients with bladder cancer with sensitivity ranging from 58–92% [8,10-12].

Here we describe a case in which a urine-based *FGFR3* genetic test, (CertNDx™ Bladder Cancer Assay [Predictive Biosciences, Lexington, MA, USA]) was pivotal in diagnosing an upper urinary tract carcinoma that was difficult to diagnose by cytology or endoscopy.

## Case presentation

**Figure 1:**
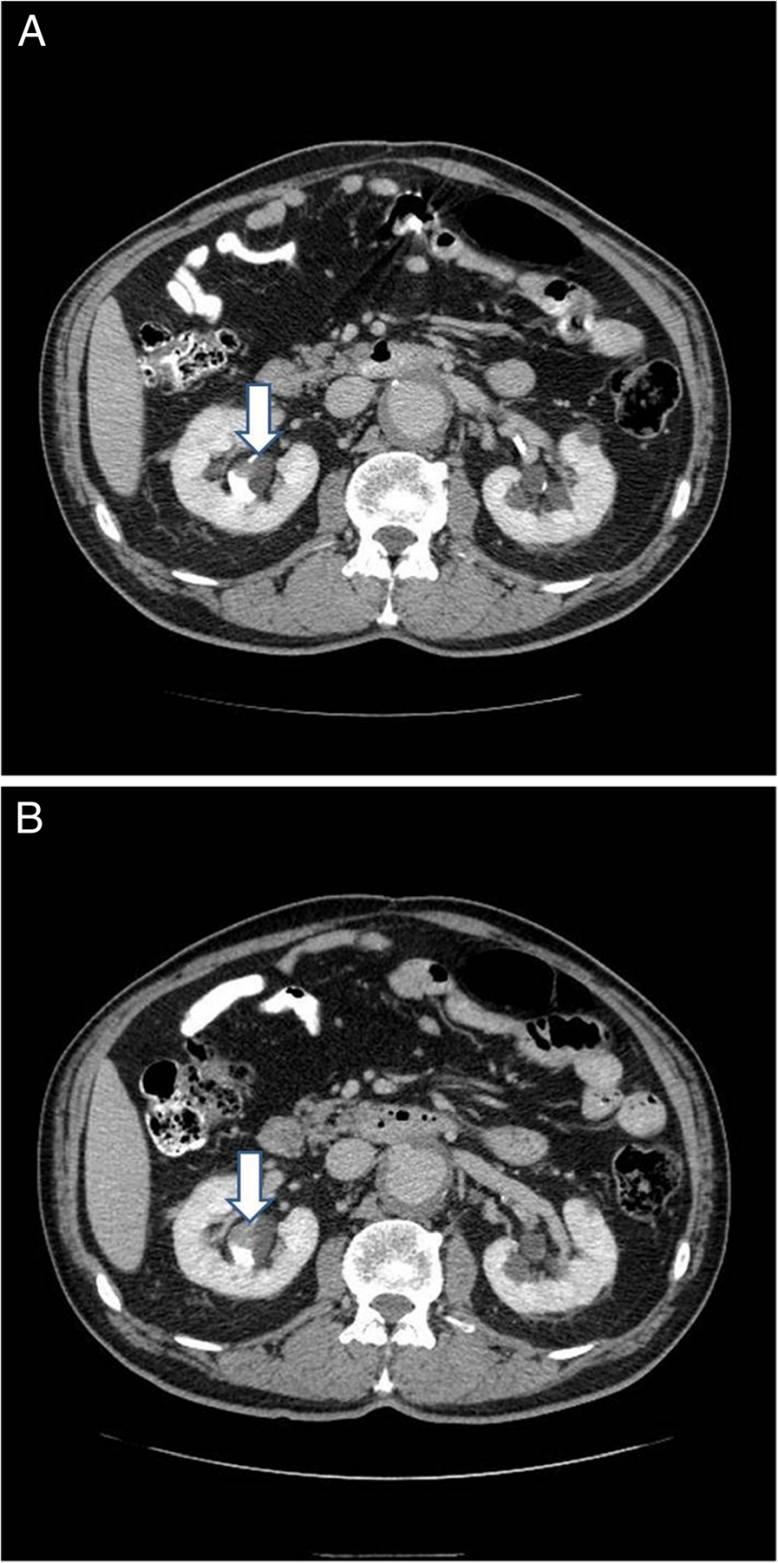
CT scans indicating the presence of the tumor (arrow) from (A) September 2010 and (B) June 2011.

A 73-year-old male presented to Urology Specialists of the Lehigh Valley in October 2010 with a right renal pelvis filling defect, potentially a urothelial carcinoma. The lesion was detected by CT scan (Figure 1A) performed for unrelated reasons and had not been visible on prior CT scans.

The patient had smoked for 20 years but had stopped smoking approximately 10 years prior. He previously had non-small cell lung cancer that was treated with radiation and chemotherapy in 2001–2002 and was in remission. In 2001–2002 he had a coronary artery bypass graft and an abdominal aortic aneurysm repaired. The patient was asymptomatic from these conditions at the time of presentation.

A physical examination was normal. The laboratory values were within normal limits. The patient had no urinary complaints. Urine cytology and cystoscopy were negative. Right retrograde pyelogram disclosed a complete ureteral duplication. Complete ureteropyeloscopy was not possible due to the narrow ureters. Retrograde pyelogram of the lower pole was performed and was normal. It was not possible to perform a retrograde pyelogram of the upper pole unit because the ureter was only about 1 mm in diameter, where a normal ureter is 3–4 mm in diameter. The instruments used in our practice are sized and scaled for a normal ureter and not for this small ancillary ureter. An attempted pyelogram was unsuccessful as the contrast did not fill the ureter or renal pelvis.

Approximately 6-months following initial urological evaluation, CT scan confirmed the presence of the mass which now appeared larger (Figure 1B). These findings were consistent with urothelial carcinoma of the renal pelvis, although urine cytology was again negative.

Prior to initiating more invasive diagnostic methods, a real-time PCR-based genetic assay was used to determine if the patient’s urine contained DNA that carried *FGFR3* mutations in exons 7, 10, or 15 [13]. This assay has 99.9% specificity for urothelial carcinoma. A mutation was detected in exon 10 (Y375C) of *FGFR3*, indicating a high probability (94.7% PPV) that the patient had urothelial carcinoma.

The patient underwent right nephroureterectomy. The arterial anatomy precluded an upper pole nephroureterectomy. The tumor involved the renal pelvis of the upper pole collecting system. Upon cut sections, the kidney exhibited an ill-defined partially raised, partially nodular tan-pink dense focus, located in the renal pelvis of the upper pole, which measured 1.5 cm greatest dimension. This focal area appeared limited to the upper pole renal pelvis/calyx and abutted but did not involve kidney parenchyma or peripelvic fat. Due to autolysis, tumor grade was somewhat difficult to provide definitively. However, the pathologist favored a designation of low grade urothelial carcinoma (WHO 2004). No lamina propria, renal parenchyma or peri-nephric fat involvement was identified such that the tumor stage was Ta,N0,M0. Tumor tissue obtained from the archival paraffin block was found, using quantitative PCR, to have an exon 10 (Y375C) mutation, which is consistent with the tumor being the source of the mutant DNA found in the urine.

Since the nephroureterectomy, the patient has been monitored for recurrent cancer. We performed a postoperative CertNDx test in March 2012, 7 months after the nephroureterectomy, which was negative for the presence of *FGFR3* mutant DNA. In addition following the uncomplicated postoperative course, the patient had surveillance cystoscopies in November 2011 and February 2012, both of which were negative. As part of the continuing follow-up, the patient will undergo surveillance cystoscopy several times per year for the foreseeable future. In view of the negative CertNDx test, upper tract imaging has not yet been performed. The left kidney has not been examined as it was normal at the time of the most recent CT scan (June 2011).

## Discussion

We describe a case in which the urine based CertNDx™ Bladder Cancer Assay, for mutations in *FGFR3*, played a pivotal role in diagnosing urothelial cancer in the urinary tract of a patient. The case was particularly difficult. A filling defect in the right renal pelvis was detected by CT scan raising the possibility that the patient had urothelial carcinoma. However, CT scans are nonspecific and filling defects are also visible in a number of other pathologies [14]. Therefore, it was important to characterize this lesion further before performing nephroureterectomy.

Diagnosis of upper urinary tract urothelial carcinoma depends heavily upon imaging and cytology. However, in this case a number of standard imaging techniques were unable to diagnose the lesion with certainty, and the urine cytology was negative. The patient had a complete ureteral duplication and the ureteral orifice to the narrow upper pole ureter was right at the bladder neck. Direct endoscopy of the upper pole moiety was attempted but proved to be impossible due to these anatomic issues. The urine cytology may have been negative due to its inherent poor sensitivity (from 4–31%) for detecting low-grade tumors [15,16].

Analysis of mutations in the *FGFR3* gene, using the urine based CertNDx™ Bladder Cancer Assay, detected cancer indicating that the tumor was malignant; which was subsequently confirmed by pathology. This assay was originally developed for monitoring patients with a history of bladder cancer. In a Phase III, multicenter, prospective study of 748 patients with hematuria and no history of bladder cancer, this assay demonstrated a specificity of 99.9% and a positive predictive value of 95.2% [13]. In a prior clinical trial of 200 patients with low-grade non-muscle-invasive bladder tumors, analysis of *FGFR3* mutations identified 3 patients with upper urinary tract recurrent cancer that were not detected by cystoscopy [10]. This study is the first to show that this assay, in a community based setting, can identify upper urinary tract urothelial carcinoma.

A number of urine based biomarkers for bladder cancer are being developed that are either protein or genetically based. The different protein assays are predicated on the observation that urine levels of certain proteins are altered when cancer is present. These assays monitor proteins that include bladder tumor antigens, nuclear matrix protein 2, bladder tumor cell-associated mucin, carcinoembryonic antigen, BLCA-1 and BLCA-4 transcription factors, matrix metalloproteinase-9, carcinoembryonic antigen-related cell adhesion molecule, survivin, or telomerase [15,17]. The genetic assays, besides *FGFR3* mutational analysis, detect chromosomal abnormalities (such as aneuploidy, etc.) using FISH (fluorescent in-situ hybridization), changes in gene methylation, and micro-RNA expression [15]. Currently, only a small number of these biomarkers are commercially available in the US, and how the others will translate into the clinic is difficult at this time to predict [15]. Presently, the *FGFR3* assay is the only urine based genetic assay that is commercially available.

## Conclusion

This case study demonstrates that the *FGFR3* assay is a beneficial addition to the methodologies used in real-life clinical settings for diagnosing not only bladder cancer but also for diagnosing the less common and more difficult to detect upper urinary tract urothelial carcinoma.

## Consent

The patient has given written consent for this case report to be published.

## Competing interests

The author received a nominal fee for his expertise and work on this case report.

## Authors' contributions

DS cared for the patient, was instrumental in the development of the manuscript, and approved the final version.

## Authors' information

Urology Specialists of Lehigh Valley, 5018 Medical Center Circle, Suite 240. Allentown, PA 18106, USA.

## Pre-publication history

The pre-publication history for this paper can be accessed here:

http://www.biomedcentral.com/1471-2490/12/20/prepub
